# MicroRNAs regulating autophagy: opportunities in treating neurodegenerative diseases

**DOI:** 10.3389/fnins.2024.1397106

**Published:** 2024-11-08

**Authors:** Mahdi Mohseni, Ghazal Behzad, Arezoo Farhadi, Javad Behroozi, Hamraz Mohseni, Behnaz Valipour

**Affiliations:** ^1^School of Medicine, Shahid Beheshti University of Medical Sciences, Tehran, Iran; ^2^School of Medicine, Tehran University of Medical Sciences, Tehran, Iran; ^3^Department of Genetics and Molecular Medicine, School of Medicine, Zanjan University of Medical Sciences, Zanjan, Iran; ^4^Department of Medical Genetics, Faculty of Medical Sciences, Tarbiat Modares University, Tehran, Iran; ^5^School of Medicine, Shahid Beheshti University of Medical Sciences, Tehran, Iran; ^6^Department of Basic Sciences and Health, Sarab Faculty of Medical Sciences, Sarab, Iran; ^7^Department of Anatomical Sciences, Faculty of Medicine, Tabriz University of Medical Sciences, Tabriz, Iran

**Keywords:** microRNA, autophagy, neurodegenerative diseases, therapeutic interventions, autophagy-related genes

## Abstract

Neurodegenerative diseases (NDs) are increasingly prevalent in our aging population, imposing significant social and economic burdens. Currently, most ND patients receive only symptomatic treatment due to limited understanding of their underlying causes. Consequently, there is a pressing need for comprehensive research into the pathological mechanisms of NDs by both researchers and clinicians. Autophagy, a cellular mechanism responsible for maintaining cellular equilibrium by removing dysfunctional organelles and misfolded proteins, plays a vital role in cell health and is implicated in various diseases. MicroRNAs (miRNAs) exert influence on autophagy and hold promise for treating these diseases. These small oligonucleotides bind to the 3’-untranslated region (UTR) of target mRNAs, leading to mRNA silencing, degradation, or translation blockade. This review explores recent findings on the regulation of autophagy and autophagy-related genes by different miRNAs in various pathological conditions, including neurodegeneration and inflammation-related diseases. The recognition of miRNAs as key regulators of autophagy in human diseases has spurred investigations into pharmacological compounds and traditional medicines targeting these miRNAs in disease models. This has catalyzed a new wave of therapeutic interventions aimed at modulating autophagy.

## 1 Introduction

The hallmark pathological feature observed in several neurodegenerative disorders, including Alzheimer’s disease (AD), Parkinson’s disease (PD), Huntington’s disease (HD), and amyotrophic lateral sclerosis (ALS), is the accumulation of misfolded protein aggregates (Aguzzi and O’connor, 2010). These protein aggregations, found in diverse cellular environments and subcellular compartments, are frequently associated with the pathological manifestations observed in various neurodegenerative diseases. Genetic mutations, leading to either autosomal recessive or dominant familial forms, can underlie the development of neurodegenerative diseases. Moreover, disruptions in proteostasis mechanisms may also contribute to the buildup of protein aggregates in these conditions (Guo et al., 2018).

Autophagy has emerged as a pivotal player in neuronal function and the pathogenesis of neurodegenerative diseases, supported by multiple lines of evidence demonstrating its ability to clear protein aggregates (Nah et al., 2015). In various neurodegenerative disorders, the accumulation of proteins within neurons is typically targeted for degradation through autophagy (Ravikumar et al., 2002; Menzies et al., 2015). Dysfunction in autophagy has been implicated as a contributing factor in the onset of many neurodegenerative disorders, particularly due to their association with genetic mutations, further underscored by the roles of disease-associated genes (Menzies et al., 2015). Autophagy’s role in safeguarding the nervous system is complex, as evidenced by a growing body of literature (Puyal et al., 2012). Its primary function is to mitigate the degeneration of post-mitotic neurons and enhance their survival (Levine and Kroemer, 2008; Son et al., 2012). Axonal damage results in the aggregation of autophagosomes and dystrophic swelling, with synapses being particularly susceptible to autophagic degradation due to their high energy and protein demands (Son et al., 2012). Moreover, heightened autophagy has been linked to increased cellular mortality, with research suggesting that excessive and prolonged activation of autophagy can culminate in self-destructive outcomes (Puyal et al., 2012; Martinet et al., 2009; Renna et al., 2010). Dysregulation of autophagy and impairment in the protein degradation system lead to the aggregation of damaged or mutated proteins within neurons, resulting in cellular damage and ultimately neuronal demise associated with neurodegeneration (Levine and Kroemer, 2008; Son et al., 2012).

MicroRNAs (miRNAs), intrinsic noncoding RNAs spanning 18 to 25 nucleotides, are extensively distributed across diverse species and hold pivotal roles in governing cell proliferation, immune response, and homeostasis (Gunel et al., 2021; D’Adamo et al., 2017; Shademan et al., 2022). Through specific binding to sequences within the 3′-untranslated region (3′-UTR) of target genes, miRNAs can finely tune the expression of nearly 30% of protein-coding genes. This regulatory mechanism can prompt mRNA cleavage or translational inhibition, ultimately eliciting considerable changes in protein levels (Shademan et al., 2023a; Wang X. et al., 2017; Shademan et al., 2023b). Furthermore, a solitary miRNA can orchestrate a genetic network by regulating multiple target genes, thereby imparting significant cumulative effects on gene networks and influencing a plethora of biological processes and diseases. The treatment potential for neurological disorders hinges on the modulation of autophagy by miRNAs. For instance, miR-144 triggers the inhibition of mTOR and initiates autophagy in response to hemoglobin in microglial cells from the hippocampus of rats (Wang Z. et al., 2017). Moreover, the decrease in mTOR levels via miR-144 exacerbates brain damage and enhances pro-inflammatory responses in mouse models of intracerebral hemorrhage, suggesting the miR-144/mTOR pathway as a promising target for intracerebral hemorrhage (ICH) treatment (Yu et al., 2017). Beyond the mTOR-dependent pathway, miRNAs also regulate other pathways relevant to aging-related neurological disorders. In Alzheimer’s Disease (AD) patients and mouse models, a non-mTOR pathway has been identified, where miRNA-regulated autophagy relies on MAPK. In AD models, inhibiting miR-101a promotes MAPK1-mediated autophagy, potentially playing a pivotal role in neurodegeneration (Li Q. et al., 2019). The utilization of miRNA-mediated regulation of autophagy holds potential for treating AD and other disorders, similar to the therapeutic effects demonstrated by resveratrol (Kou and Chen, 2017).

MicroRNAs, integral components of noncoding RNA, intricately regulate all phases of autophagy. A notable example involves the transcription factor c-MYC, which governs diverse cellular processes such as cell growth, proliferation, and apoptosis. In a study conducted by Lu et al., patients with Crohn’s disease displayed elevated levels of c-Myc, leading to heightened expression of miR106B and miR93. Consequently, this dysregulation resulted in reduced autophagosome formation and impaired clearance of intracellular bacteria via targeting ATG16L1 (Lu et al., 2014). Moreover, miR-376b, miR-17-5p, miR-216a, and miR-30a/b downregulated the expression of BECLIN1, impeding the initiation phase of vesicle formation. MiR-204 directly targeted LC3, hindering the elongation stage, while ATG4 was modulated by miR-101, miR-34a, miR-24-3p, and miR-376b (Tu et al., 2019). Notably, macrophages may undergo impaired autophagosome maturation due to the upregulation of miR-423-5p, which suppresses the fusion of autophagosomes and lysosomes (Li et al., 2016). Subsequent sections will delve into the intricate mechanisms by which microRNAs regulate autophagy across various disease phenotypes. By targeting specific genes or modulating autophagy-related signaling pathways, microRNAs have the potential to either enhance or suppress autophagy. The significance of microRNAs in autophagy regulation extends to their utility as both diagnostic and prognostic markers.

## 2 Autophagy types and process

Autophagy, an essential cellular process, ensures internal equilibrium by producing autophagosomes, which are double-membrane vesicles that encapsulate long-lasting proteins or organelles such as mitochondria. Subsequently, these autophagosomes ferry the captured material to lysosomes for degradation (Boland and Nixon, 2006). Despite the presence of autophagy machinery across various species, the brain possesses specific mechanisms to regulate its nutrient and energy supply, leading to the relatively late recognition of basal autophagic flux in healthy neurons (Boland and Nixon, 2006). The initial evidence showcasing the significance of autophagy in the brain likely arose from the discovery of knockout mice lacking autophagy-related proteins 5 and 7 (Atg5, Atg7) (Hara et al., 2006; Komatsu et al., 2006). The absence of Atg7 resulted in notable neuronal depletion in the cerebral and cerebellar cortices, accompanied by observable behavioral impairments and an accumulation of polyubiquitinated proteins within neurons (Komatsu et al., 2006). The acknowledgment of autophagy’s pivotal role in maintaining brain health is now widespread, fueling a surge in literature on this topic. Numerous human neurodegenerative diseases are characterized by autophagy dysfunction, arising from gene mutations or the accumulation of potentially toxic proteins prone to aggregation. The clearance of autophagic organelles, particularly damaged mitochondria (mitophagy), is currently a focal point of research, given the brain’s distinct reliance on energy and the suspicion that this process may be compromised in various pathologies (Van Laar and Berman, 2013).

Autophagy, the cellular process of component degradation, is classified into three types based on how cargo reaches lysosomes in mammals: chaperone-mediated autophagy, microautophagy, and macroautophagy. Collectively, they are referred to as autophagy for simplicity (Park et al., 2020). Autophagosomes, specific double membrane-bound vesicles, envelop unnecessary or misfolded proteins and damaged subcellular organelles, which are subsequently transported to lysosomes for breakdown. To maintain cellular function, mTORC1 restricts autophagy to a minimum level in most cells (Park et al., 2020). However, under various forms of cellular stress like nutrient deprivation, growth factor withdrawal, or oxygen depletion, autophagy is triggered by the release from mTORC1 inhibition, leading to a significant increase to meet heightened energy demands.

Autophagy is a multi-step process governed by specific complexes exclusively dedicated to autophagy, meticulously controlled by upstream signaling molecules. The activity of autophagy is regulated in contrasting manners by mTORC1 and AMP-activated protein kinase (AMPK), the two primary signaling molecules. This regulation occurs through the phosphorylation of Unc-51 like autophagy activating kinase 1 (ULK1) at distinct sites. Autophagy initiation is inhibited by mTORC1 through the phosphorylation of ULK1 at Ser757, while AMPK stimulates autophagy initiation by phosphorylating ULK1 at Ser317, Ser555, and Ser777 (Di Nardo et al., 2014; Jang et al., 2018; Kim et al., 2011). The autophagy preinitiation complex, ULK1, consists of the ULK1 protein kinase, FIP200/RB1CC1, and regulatory subunits ATG101 and ATG13. These subunits induce conformational changes that activate ULK1 (Lin and Hurley, 2016; Turco et al., 2020). Upon activation, the ULK1 complex phosphorylates the VPS34 complex, a downstream autophagy initiation complex comprising Beclin-1, VPS34, VPS15, and ATG14L, belonging to the class III phosphoinositide 3-kinase (PI3K) family (Mercer et al., 2021). PI3P is generated by the VPS34 complex on specific phospholipid membranes, including the endoplasmic reticulum (ER), ER-mitochondria junctions, and ER-plasma membrane connections (Zhen and Stenmark, 2023). Proteins that bind to PI3P, such as ZFYVE1/DFCP1 or WD repeat domain-containing proteins (WIPIs), are recruited to membrane structures rich in PI3P, termed omegasomes. Subsequently, they recruit autophagy-related proteins, culminating in the formation of the phagophore structure (Puri et al., 2018; Dooley et al., 2014).

Recent research has shed light on the complex regulatory mechanisms governing the expansion of the phagophore. Key autophagy proteins, notably ATG9A, a transmembrane protein, play central roles in this process. Upon autophagy activation, ATG9A relocates from the trans-Golgi network (TGN) or endocytic compartments to the omegasomes, a process regulated by either ULK1 or the retromer complex (Popovic and Dikic, 2014; Zhou C. et al., 2017; Young et al., 2006). ATG9A-containing vesicles are crucial for autophagosome formation as they translocate to the outer membrane, indicating their function as providers of lipid bilayers in this process (Olivas et al., 2023). The interaction between the phagophore and ATG2 protein is facilitated by ATG18’s ability to bind to PI3P (Rogov et al., 2023). Regulation of the size of initial autophagic structures requires the attachment of the PI3P-enriched membrane through the ATG2-ATG18 complex (Chowdhury et al., 2018). Additionally, the yeast Atg2 protein is reported to play a vital role in autophagy through its lipid transfer function (Osawa et al., 2019). Although the precise mechanisms of autophagosome closure remain elusive, there is mounting evidence implicating ATG2, VPS21, and the endosomal sorting complexes required for transport (ESCRT) complex in this process (Takahashi et al., 2018; Lee et al., 2007; Zhou F. et al., 2017).

## 3 Functional roles of autophagy in neurodegenerative diseases

Neurodegenerative disorders often manifest with abnormal protein accumulations, leading to the formation of neurofibrillary tangles. Examples of these proteins include amyloid precursor protein (APP) Aβ and C-terminal fragments (CTF) in Alzheimer’s disease (AD), mutant α-synuclein in Parkinson’s disease (PD), and polyglutamine (polyQ)-expanded mutant HTT (mHtt) in Huntington’s disease (HD) (Shademan et al., 2021; Chen et al., 2024; Deng et al., 2017; Menzies et al., 2017). The autophagy-lysosome degradation pathway primarily targets protein aggregates found in neurodegenerative diseases. Genetic mutations in autophagic receptors such as p62, OPTN, NBR1, and ALFY/WDFY3 have been frequently linked to neurodegenerative diseases (Deng et al., 2017; Scrivo et al., 2018). Autophagic activity declines significantly with aging, which is the most common risk factor for neurodegeneration (Stavoe et al., 2019). Impaired autophagy is believed to contribute to the onset of neurodegenerative disorders.

### 3.1 Alzheimer’s disease (AD)

Alzheimer’s disease (AD) is the leading cause of neurodegenerative dementia. Its characteristic features include the accumulation of Aβ plaques and tau neurofibrillary tangles in the brain, which are considered central to its pathogenesis. Aβ, a peptide derived from amyloid precursor protein (APP) processing, is primarily cleaved by α-, β-, and γ-secretase in the trans-Golgi network (TGN) and endosomes (Hoseinlar et al., 2023; Radagdam et al., 2023). Autophagy serves as the primary mechanism for clearing Aβ and APP-CTF (Lee et al., 2019; Uddin et al., 2018). Enhanced activity of p62 or transcription factor EB (TFEB) has been demonstrated to reduce Aβ plaque formation, thus ameliorating AD pathology in mouse models (Song et al., 2020). Conversely, elevated Aβ oligomers in animal models induce impairments in trafficking and lysosome biogenesis, leading to hindrances in autophagic activity (Tammineni et al., 2017). Examination of AD patient brains at the ultrastructural level reveals the accumulation of autolysosomes containing cathepsin due to defects in lysosomal proteolysis (Nixon and Yang, 2011; Boland et al., 2008). Moreover, alterations in the levels of autophagy-related proteins are commonly observed in samples from AD patients (Lachance et al., 2019; Qian et al., 2022). Autophagy also participates in the degradation of abnormally phosphorylated tau (Lee et al., 2019). Post-mortem analysis of AD patient brains shows protein accumulation in the autophagy-lysosomal pathway, including p62, LC3, and LAMP1, along with disruptions in both autophagy and lysosomal processes (Piras et al., 2016). Hyperphosphorylated tau directly interacts with aggrephagy receptors such as p62, NDP52, and OPTN, facilitating its subsequent removal through autophagic degradation (Xu et al., 2019; Jo et al., 2014). PICALM is involved in the regulation of tau’s autophagic degradation. Impaired transportation of the dynein-dynactin complex, essential for autophagosome movement, leads to an increase in tau-positive filaments (Butzlaff et al., 2015). Conversely, activation of autophagy accelerates the breakdown of phosphorylated tau, preventing its aggregation both in vitro and in vivo (Krüger et al., 2012; Wang et al., 2009).

### 3.2 Parkinson’s disease (PD)

Parkinson’s disease (PD) is a progressive movement disorder affecting the nervous system, characterized by the presence of Lewy bodies in dopaminergic neurons of the substantia nigra, which are abnormal aggregations of α-synuclein protein. Additionally, there is an elevation in the expression of α-synuclein genes (Singleton et al., 2003). Knockout of ATG7 led to an increase in the formation of α-synuclein inclusion bodies containing p62 in dopaminergic neurons, along with age-related motor function impairments in mice (Sato et al., 2018). Several studies indicate that the autophagy-lysosome system is responsible for degrading α-synuclein with pathogenic mutations (Yan Yuan et al., 2018; Vogiatzi et al., 2008). Conversely, α-synuclein inclusions impair the autophagic pathway at multiple stages. For instance, α-synuclein inclusions disrupt the formation of omegasomes by misplacing ATG9A (Winslow et al., 2010; Tanik et al., 2013). Moreover, α-synuclein clustering hampers the retrograde movement of autophagosomes, although it does not inhibit autophagosome-lysosome fusion (Tanik et al., 2013). Ultimately, α-synuclein interferes with autophagic degradation and the activity of the lysosomal protease cathepsin D (CTSD) (Hoffmann et al., 2019; Moors et al., 2019). Research suggests that LRRK2 deficiency inhibits the autophagy-lysosome pathway, leading to cell death (Tong et al., 2010). Notably, many pathogenic LRRK2 mutations are considered gain-of-function mutations, such as G2019S and R1441C (Kett and Dauer, 2012). These mutations enhance LRRK2 kinase activity but impair autophagic degradation, resembling the effects of LRRK2 deficiency (Plowey Cherra et al., 2008; Ramonet et al., 2011). Studies have shown that the LRRK2-G2019S mutation disrupts endocytic vesicular trafficking by reducing small GTPase activity, while the LRRK2-R1441C variant compromises lysosomal functions due to impaired interaction with the lysosomal v-ATPase (Wallings et al., 2019). Mutations in VPS35, a key component of the retromer complex, have been associated with impaired autophagy in PD. VPS35 is crucial for regulating the transport of lysosomal proteases (Miura et al., 2014). Reduced mRNA levels of VPS35 were observed in the substantia nigra of PD patients (MacLeod et al., 2013). The presence of a PD-related VPS35 mutation (D620N) within a family hindered the recruitment of the WASH complex to endosomes, leading to malfunction in ATG9A positioning and impaired autophagy (Zavodszky et al., 2014).

### 3.3 Huntington’s disease (HD)

Huntington’s disease (HD) is an autosomal-dominant progressive neurodegenerative disorder characterized by neuronal degeneration, leading to motor, behavioral, and cognitive impairments. The striatum and cortex are affected by cytotoxicity caused by an abnormal expansion of a polyQ repeat in exon 1, resulting in the formation of mutant huntingtin (mHtt) proteins that form β-sheet-rich structures and ubiquitin-positive aggregates (Takeuchi and Nagai, 2017; Maat-Schieman et al., 1999). Overexpression of mHtt leads to progressive motor deficits and accumulation of autophagosomes (Pircs et al., 2018), with an observed increase in autophagic vacuoles in HD patients (Oh et al., 2022). Autophagy plays a crucial role in clearing proteins prone to aggregation with polyQ expansion, both in vitro and in vivo (Wu et al., 2012; Proenca et al., 2013). Inhibition of autophagy, either through autophagy inhibitors like 3-MA or Baf.A1 or genetic manipulation, increases mHtt aggregation, while administration of autophagy activators like rapamycin or trehalose reduces inclusion body count. The turnover rate of mHtt is influenced by its interaction with aggrephagy receptors such as p62 and OPTN (Fu et al., 2017; Tsvetkov et al., 2013). Genome-wide analysis focused on the striatum has identified numerous autophagy-related genes, including Atg4b, Tfeb, and Atlastin 3, which seem to mitigate mHtt toxicity (Wertz et al., 2020). Huntingtin typically acts as a scaffold protein for various autophagy proteins, aiding in cargo identification and protecting against Beclin-1 ubiquitination (Ashkenazi et al., 2017). However, mHtt fails to recognize cytosolic cargoes present in autophagosomes (Martinez-Vicente et al., 2010). Additionally, huntingtin knockdown or mHtt overexpression in neurons may impair autophagosomal retrograde transport (Wong and Holzbaur, 2014).

## 4 Review on MicroRNAs

MicroRNAs (miRNAs) are essential small non-coding RNA molecules that play a pivotal role in regulating gene expression. Their production encompasses several steps. Initially, miRNA genes undergo transcription by either RNA polymerase II or RNA polymerase III, resulting in primary miRNA (pri-miRNA), a lengthy transcript (Gunel et al., 2021). Pri-miRNA adopts a stem-loop structure with a 5′ cap and a poly-A tail. Ribonuclease enzyme Drosha, aided by DGCR8, cleaves pri-miRNA to generate pre-miRNA, a hairpin-like structure of approximately 60-100 nucleotides (Shademan et al., 2022; Ma et al., 2018). Ran GTP and exportin-5 facilitate the transportation of pre-miRNAs from the nucleus to the cytoplasm. Upon entering the cytoplasm, hydroxylation of Ran GTP transforms it into Ran GDP, releasing pre-miRNA from exportin 5 (Ohtsuka et al., 2015). In the cytoplasm, Dicer, along with a protein partner, cleaves pre-miRNA to produce mature miRNA, a double-stranded molecule of about 22 nucleotides without a hairpin structure (Ohtsuka et al., 2015; Fu et al., 2019). The miRNA/miRNA* duplex may contain unpaired bases and incomplete bonding. Subsequently, one strand of the duplex, either miRNA or miRNA*, integrates into the RNA-induced silencing complex (RISC), guiding mature miRNA to its target transcript, thereby inhibiting translation and suppressing protein synthesis (Krol et al., 2004; Shademan et al., 2023a).

The discovery of miRNAs has illuminated their involvement in regulating up to 200 mRNAs, representing approximately 1% of the human genome (Chen et al., 2012). Understanding miRNA function and biogenesis is vital for comprehending their roles in various biological processes such as development, cell differentiation, and disease. Investigating miRNA functions involves identifying their target genes and the biological pathways they partake in, utilizing methodologies like microarray analysis and RNA sequencing (Gon et al., 2020).

MiRNAs play a crucial role in regulating cell growth, proliferation, and homeostasis in the human brain, with their expression exhibiting variability. Specific miRNAs modulate genes associated with neurodegenerative disorders (NDs) (Chen et al., 2018; Adlakha and Saini, 2014). Animal models have demonstrated that Dicer, an essential RNase, triggers the production of adult miRNAs crucial for neuronal survival in the cerebral cortex. Dicer deficiency leads to impaired neurogenesis and significant neuronal loss (Adlakha and Saini, 2014; Hollins and Cairns, 2016). The absence of Dicer results in compromised neurogenesis and widespread neuronal loss. Notably, neuronal cell counts decrease in the hippocampus, tau proteins undergo hyperphosphorylation, and neurodegenerative symptoms emerge in the adult brain in the absence of Dicer (Sharma and Lu, 2018). Understanding the role of miRNAs in normal cellular processes and their dysregulation leading to neurological disorders is pivotal for developing innovative treatments for NDs.

Numerous miRNAs play a crucial role in regulating cognitive functions and preventing memory loss in Alzheimer’s disease (AD) by maintaining protein-mediated activity at the synaptic level (Kou et al., 2020). Research indicates that miRNAs modulate the expression of genes involved in amyloid beta (Aβ) formation, tau phosphorylation, and neuroinflammation in AD. MiRNAs such as miR-29a/b, miR-135a, miR-124, and miR-195 target β-secretase (BACE1) and regulate Aβ production (Gentile et al., 2022). Individuals with Parkinson’s disease (PD) exhibit reduced expression of MiR-150. Overexpression of MiR-150 in BV2 cells treated with LPS inhibits the release of TNF-α, IL-1β, and IL-6 (Li et al., 2020). MiRNAs including Let7, miR-10a/b, miR-181, miR-182, and miR-212 regulate the expression of alpha-synuclein, implicated in PD onset (Recasens et al., 2016). Furthermore, decreased levels of miRNA 9 in the high-definition brain amplify REST transcription, resulting in enhanced repression of BDNF in neurons. Various miRNAs, such as miRNA 29a/b, miRNA 124a 1/2/3, miRNA 132, miRNA 135b, miRNA 139, miRNA 212, and miRNA 346, target REST (Gupta et al., 2015). Understanding the biogenesis process and function of miRNAs is crucial to grasp their impact on gene regulation and various biological processes. This understanding could have significant implications for disease detection, treatment, and prevention.

## 5 Autophagy regulation by miRNAs in human neurodegenerative diseases

MiRNAs play a significant role in modulating autophagy-related genes and signaling pathways. Changes in the expression patterns of these miRNAs can influence the pathological progression of neurodegenerative diseases like Alzheimer’s disease (AD), Parkinson’s disease (PD), and Huntington’s disease (HD) by affecting autophagy. Hence, our research focuses on understanding how miRNAs impact autophagy in these conditions. Table 1 provides a list of miRNAs known to regulate autophagy in neurodegenerative diseases.

**TABLE 1:** Some studies that investigated miRNAs involved in autophagy in neurological diseases.

miRNAs	Experimental model	miRNA status in ND	Results on autophagy	Target	Disease	Clearance mechanism	References
miR-34a	SH-SY5Y cells	Upregulated	Down-regulation of miR-34a inhibits autophagy	DRP1, MFN2	AD	Abnormal mitochondrial dynamics	Kou et al., 2017
miR-130a	SH-SY5Y cells	Downregulated	Upregulation of miR-130a Induced autophagy	LC3, Ac-p53, p21 and p62	AD	Up-regulating autophagy	Shen et al., 2021
miR-34c-5p	SH-SY5Y cells	Downregulated	Upregulation of miR-34c-5p suppresses autophagy	ATG4B, LC3-II	AD	Suppresses autophagy	Dai et al., 2018
miR-34a	Model rats with D-gal-induced brain	Upregulated	The inhibition of miR-34a triggers autophagy via the SIRT1 and mTOR signaling pathways.	LC3, p62 and Atg7	AD	Rescued defective autophagy	Kou et al., 2016
miRNA-101a	APPswe/PS1DE9 transgenic mice	Downregulated	Elevated levels of miRNA-101a can modulate the process of autophagy formation.	65 genes are associated with AD	AD	Autophagy phenomenon regulated	Li Q. et al., 2019
miR-135a-5p	SH-SY5Y and CHP 212 cells	Downregulated	Overexpression of miR-135a-5p can inhibition of autophagy	mTOR/ULK1/S6K1	PD	Silencing suppressed autophagy	Qin et al., 2022
miR-106b	mice and mouse primary	Downregulated	Increased expression of miR-106b can activate autophagy.	CDKN2B, LC3-II	PD	Enhance neuronal autophagy	Bai et al., 2021
miR-199a	PC12 cells	Downregulated	Elevated levels of miR-199a can inhibit autophagy.	PTEN/AKT/mTOR, LC3-II, Beclin-1	PD	Autophagy-regulating	Ba et al., 2020
miR-326	mice	Downregulated	Increased expression of miR-326 can activate autophagy.	XBP1/JNK, LC3-II	PD	Promotes autophagy of dopaminergic neurons	Zhao et al., 2019
miR-212-5p	mice and SH-SY5Y cells	Downregulated	Elevated levels of miR-212-5p can activate autophagy.	SIRT2, p53 LC3-II, p62,	PD	Promoted autophagy in PD model	Sun et al., 2018
miR-181a	SK-N-SH cells	Downregulated	Elevated levels of miR-181a result in the suppression of autophagy.	p38 MAPK/JNK, Beclin-1, LC3-II	PD	Regulates autophagy in PD	Liu et al., 2017

ND, Neurodegenerative diseases; DRP1, dynamin-related protein 1; MFN2, mitofusin 2; D-gal, D-galactose; SIRT1, silent information regulation 1.

### 5.1 Alzheimer’s disease (AD)

**FIGURE 1:**
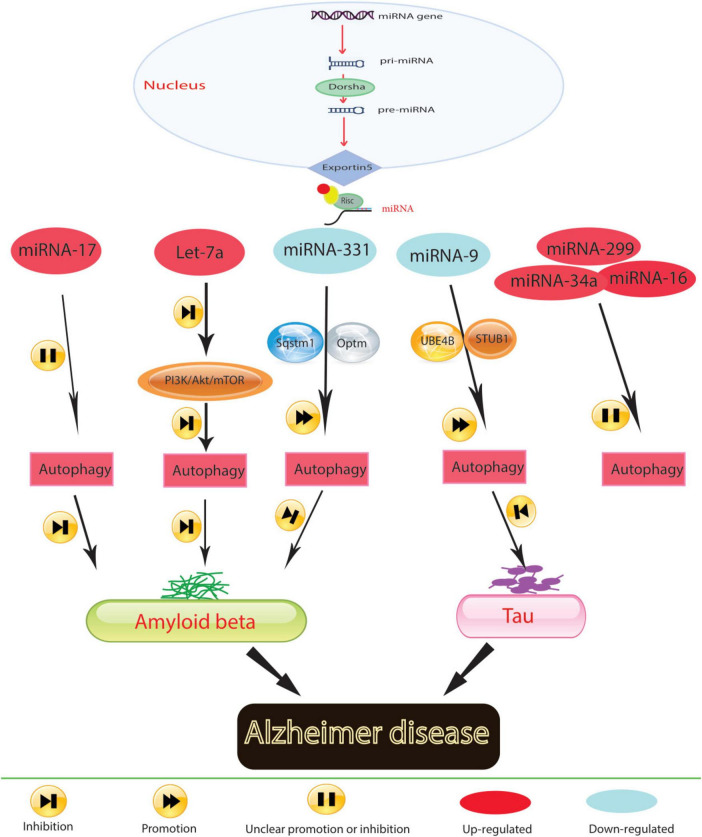
In Alzheimer’s disease, molecular mechanisms govern the role of miRNA in regulating autophagy.

During protein translation, miRNAs play a significant role in regulating autophagy-related genes, as illustrated in Figure 1. Aberrant miRNA regulation can worsen the onset and progression of Alzheimer’s disease (AD) by affecting autophagy-related proteins. Research indicates that inhibiting miR-140, which activates PINK1-mediated mitophagy, can notably reduce the incidence of AD (Liang et al., 2021). Conversely, overexpression of miR-101a indirectly induces autophagy in AD by modulating mitogen-activated protein kinase (MAPK), suggesting the regulatory function of miRNA-controlled autophagy in this disorder. Timely clearance of Aβ and tau proteins in AD models is crucial for alleviating AD symptoms. Studies suggest that up-regulating miR-9-5p can target ubiquitination factor E4B (UBE4B) and stress-induced phosphoprotein 1 (STIP1) homology and U-box containing protein 1 (STUB1), enhancing autophagy and facilitating tau protein degradation, thus providing relief from AD symptoms (Subramanian et al., 2021). In the early stages of AD, miR-331-3p and miR-9-5p exhibit a significant decrease in the APP/PS1 mouse model, while in later stages, both miRNAs sequentially increase, accompanied by abnormal functional changes in autophagy. Downregulating miR-331-3p and miR-9-5p, which target SQSTM1/p62 and OPTN, critical autophagy-related proteins, may accelerate Aβ clearance and enhance cognitive capacity (Chen et al., 2021).

Mitochondrial function is also implicated in AD pathogenesis. Elevated levels of miR-204 in AD models exacerbate reactive oxygen species generation and inhibit mitophagy by suppressing transient receptor potential mucolipin 1 (TRPML1) activity. Conversely, reducing miR-204 expression can reverse this effect (Zhang et al., 2021b). Additionally, the study results demonstrated that miR-140 was up-regulated and PINK1 was down-regulated in AD model rats and neurons. It was confirmed that PINK1 is a direct target of miR-140. Silencing miR-140 in these models mitigated mitochondrial dysfunction and enhanced autophagy. This was evidenced by decreased levels of mTOR expression and phosphorylation, β-amyloid, phosphorylated Tau at Ser396 and Thr231, total Tau, and reactive oxygen species, as well as increased mitochondrial membrane potential, Beclin 1 expression, and the LC3-II/LC3-I ratio. Thus, inhibiting miR-140 promoted autophagy and prevented mitochondrial dysfunction by upregulating PINK1 (Liang et al., 2021). MiRNAs play a conserved role in regulating autophagy across neurodegenerative diseases. In Alzheimer’s disease, miR-140 and miR-101a are notable for their effects on mitochondrial function and protein clearance, respectively. These miRNAs modulate key autophagic pathways, such as PINK1-mediated mitophagy and MAPK signaling, pointing to a potential unifying mechanism where miRNAs maintain cellular homeostasis via autophagy across different stages of neurodegeneration.

Neuroinflammation is closely associated with AD pathogenesis, highlighting the importance of targeting neuroinflammation as a therapeutic strategy. MiR-223 can mitigate neuroinflammation by regulating Atg16L1, emphasizing Atg16l1’s role in controlling autophagy and inflammation in AD individuals (Li Y. et al., 2019). The progression of AD is also influenced by neuronal function in the hippocampus. Reversing decreased levels of miR-16-5p in hippocampal tissues of AD mice inhibits neuronal apoptosis, increases neuronal viability, and improves neurological function and deficits (Dong et al., 2021). Similarly, suppressing miR-299-5p leads to increased autophagic activation, decreased apoptosis, and improved cognitive function in AD mice (Zhang et al., 2016).

Prolonged exposure to Aβ has been shown to disrupt autophagy in microglia; however, the underlying cellular changes in response to Aβ that lead to this disruption remain unknown (Pomilio et al., 2020). Members of the Mirc1/Mir17-92 cluster are known to target essential autophagy molecules (Tazi et al., 2016). The diminished expression of an individual autophagy protein targeted by elevated miRs can halt autophagic function, which cannot be compensated for by the expression of other autophagy proteins (Tazi et al., 2016). The Mirc1/Mir17-92 cluster, which includes miR-17-5p, miR-18a, miR-19a, miR-20a, miR-19b-1, and miR-92a-1, is conserved among vertebrates and is associated with roles in the cell cycle, tumorigenesis, and aging (Dellago et al., 2016). Inhibiting elevated miR-17 in 5xFAD mouse microglia improves Aβ degradation, autophagy, and NBR1 puncta formation in vitro and improves NBR1 expression in vivo (Estfanous et al., 2021). Thus, by regulating the expression of autophagy-related proteins, miRNAs can potentially modulate processes such as Aβ and tau protein clearance, mitochondrial function, neuroinflammation, neuronal damage, apoptosis, and neuronal viability in AD.

### 5.2 Parkinson’s disease (PD)

**FIGURE 2:**
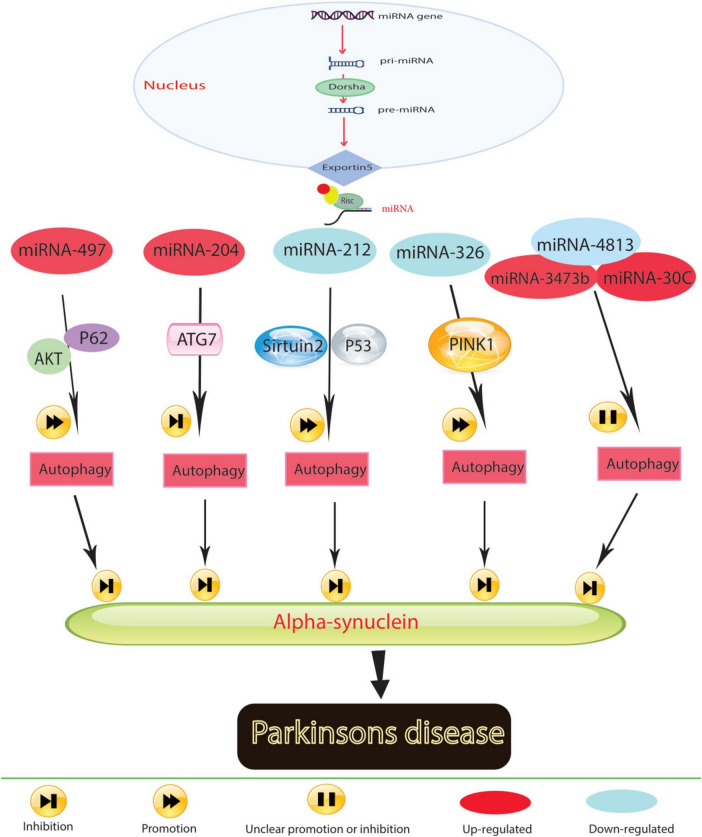
In Parkinson’s disease, molecular mechanisms govern the role of miRNA in regulating autophagy.

Research has underscored the role of miRNAs in the pathological processes of Parkinson’s disease (PD) by activating autophagy (Figure 2). For instance, the decrease in miR-326 expression levels, associated with the PD-related gene PINK1, contributes to PD progression (Choi et al., 2016). Administering a miR-326 mimic in MPTP-treated mice reduces α-synuclein and inducible nitric oxide synthase levels, improving locomotor function by enhancing autophagy in dopaminergic neurons through JNK signaling pathway activation (Zhao et al., 2019). MiR-4813-3p facilitates the clearance of clustered α-synuclein, potentially preventing neuronal oxidative damage in a transgenic Caenorhabditis elegans model of PD (Sarkar et al., 2022).

Low expression levels of miR-212-5p are observed in both SH-SY5Y cells and PD animal models. Introducing miR-212-5p mimics mitigates dopaminergic neuron loss by inhibiting sirtuin2, fostering autophagy, and reducing cytoplasmic p53 expression (Sun et al., 2018). Reduced miR-124 levels in MPP+-treated SH-SY5Y cells and MPTP-treated mice lead to autophagosome accumulation and lysosome depletion. Restoring miR-124 with agonists and mimics reduces dopaminergic neuron loss and increases striatal dopamine levels by restoring impaired autophagy and suppressing BIM expression (Wang et al., 2016). The results indicated that co-culturing injured HT22 neurons with miR-124-3p overexpressing BV2 microglia exerted a protective effect by inhibiting autophagy in the scratch-injured neurons (Li D. et al., 2019). The unique role of miR-124 in mediating the microglial inflammatory response by targeting p62 and p38 has been highlighted in PD. In the microglial culture supernatant transfer model, knockdown of p62 in BV2 cells prevented apoptosis and death of human neuroblastoma cell lines (SH-SY5Y) following microglial activation. The study results suggest that miR-124 can inhibit neuroinflammation during PD development by targeting p62, p38, and autophagy, indicating that miR-124 could be a potential therapeutic target for regulating the inflammatory response in PD (Yao et al., 2019).

Elevated miR-204-5p levels disrupt ATG7-regulated autophagy, promoting cell death and triggering JNK-mediated apoptosis in dopaminergic cells (Chiu et al., 2019). The miR-30c-5p/ATG5 relationship exacerbates PD progression by reducing antioxidants and dopamine levels, leading to neural cell apoptosis and worsening MPTP-induced motor deficits in mice (Zhang et al., 2021a). Conversely, increased miR-497-5p levels protect MPP+-treated SH-SY5Y cells from apoptosis by triggering autophagy through fibroblast growth factor-2, which regulates p62 via the AKT pathway (Zhu et al., 2021; Zhang et al., 2013). However, elevated miR-3473b levels in MPTP-treated mice stimulate microglial secretion of inflammatory substances by hindering autophagy, exacerbating the inflammatory response in PD (Lv et al., 2021). Beyond regulating autophagy, miRNAs hold promise as diagnostic biomarkers due to their disease-specific expression profiles. MiRNAs like miR-140 in Alzheimer’s and miR-326 in Parkinson’s are prime candidates for therapeutic targeting. By restoring their normal expression levels, it may be possible to halt disease progression by enhancing autophagic clearance of toxic proteins, offering a tailored therapeutic approach. These findings suggest that decreased miRNA expression in PD models is associated with α-synuclein accumulation, oxidative stress, neuronal cell death, and inflammation in the brain. Conversely, increasing miRNA levels stimulates autophagy, counteracting these harmful effects. Controlling autophagy through miRNAs holds promise as an effective therapeutic strategy for PD.

### 5.3 Huntington’s disease (HD)

Autophagy, along with other pathways for protein degradation, undergoes tight regulation by various miRNAs, underscoring their pivotal role in modulating autophagic processes within the neuronal system. Essential autophagy-related proteins like Sequestosome 1, Optineurin, BACE1, and ATG5 are directly influenced by miRNAs, thereby affecting autophagic activity (Chen et al., 2021; Zhou et al., 2021). Elevated levels of Argonaute-2 (AGO2), a crucial component of the RNA-induced silencing complex responsible for executing miRNA functions, lead to changes in miRNA abundance and effectiveness. In neurons expressing mutant huntingtin (mHtt), the presence of mature miRNAs coincides with AGO2 accumulation. However, AGO2 relocation to stress granules (SGs) induced by mHtt expression diminishes miRNA activity globally. This disparity in AGO2 accumulation has distinct effects on neurons compared to dividing cells, possibly due to neurons’ incapacity to renew their protein composition during cell division or the emergence of SGs in mHtt-expressing neurons (Pircs et al., 2018). These findings underscore the crucial involvement of miRNAs in cellular processes related to protein degradation, suggesting the potential therapeutic utility of miRNAs for various neurological disorders. Despite the promising outlook, research investigating miRNAs’ role in regulating autophagy as a treatment strategy for individuals with Huntington’s disease (HD) remains limited.

While significant strides have been made in understanding miRNA-autophagy interactions, gaps remain. Many miRNAs, such as miR-204 and miR-497-5p, have been explored primarily in animal models, with limited translation to clinical settings. Future research should aim to validate these findings in human studies and assess the long-term effects of miRNA-based therapies. Additionally, there is a need to explore the combinatorial effects of miRNA modulators with conventional treatments to optimize therapeutic outcomes. In summary, miRNA-regulated autophagy presents a multifaceted opportunity for both the diagnosis and treatment of neurodegenerative diseases. By targeting key autophagic pathways, miRNAs offer a promising route to not only slowing disease progression but also improving patient outcomes through more personalized therapeutic approaches. By integrating these changes, you can address the feedback more effectively, showing a deeper synthesis of current research, highlighting the therapeutic potential of miRNAs, and pointing out areas for future study.

## 6 Conclusion

The intersection of miRNA research and autophagy presents a promising frontier in the treatment of NDs. Given the rising prevalence of NDs and the substantial burden they place on society, there is an urgent need for novel therapeutic approaches that go beyond symptomatic relief and address the underlying disease mechanisms. Autophagy, crucial for cellular health through the removal of dysfunctional organelles and proteins, is increasingly recognized as a pivotal process in the pathophysiology of NDs. Autophagy-regulating miRNAs perform a dual function in the progression of ND. By targeting autophagy-related signaling pathways, downregulated miRNA Autophagy-controlling miRNAs serve a two-fold purpose in the development of ND. MiRNAs have a neuroprotective effect by stimulating protective autophagy or reducing autophagic cell death in neurons. Conversely, neurotoxic miRNAs increase to hinder autophagy processes. In light of this, targeting miRNA-mediated autophagy may present a promising therapeutic approach in the treatment of ND. The therapeutic potential of miRNAs in modulating autophagy opens up new avenues for treating NDs. Pharmacological compounds and traditional medicines targeting specific miRNAs have shown promise in preclinical models, offering hope for the development of effective treatments. This line of research not only enhances our understanding of the molecular mechanisms underpinning NDs but also paves the way for innovative therapeutic strategies. Continued research into miRNA-mediated autophagy regulation is essential for advancing our understanding of NDs and for the development of targeted therapies that could potentially alter the course of these debilitating diseases. The integration of miRNA research into clinical practice holds the promise of transforming ND treatment paradigms and improving patient outcomes.
